# Does a mobile interactive web application improve dental students’ panoramic radiograph interpretation skills? A pre–post educational study

**DOI:** 10.1186/s12909-026-09973-3

**Published:** 2026-07-25

**Authors:** Beyza Yalvaç Pınarbaşı, Kemal Selçuk Yücel, Deniz Ezgi Şakalakoğlu, Emin Murat Canger

**Affiliations:** 1 Kahramanmaraş Oral and Dental Health Hospital, Kahramanmaraş, Türkiye; 2 Faculty of Dentistry, Erciyes University, Kayseri, Türkiye; 3 Faculty of Dentistry, Department of Oral and Maxillofacial Radiology, Erciyes University, Kayseri, Türkiye

**Keywords:** Learning, Mobile learning, Panoramic radiography, Web-based application

## Abstract

**Background:**

This study aims to evaluate the impact of a newly developed, mobile-compatible web application on the learning process of dental interns, specifically focusing on its effectiveness in the interpretation of panoramic radiographs.

**Methods:**

The study included 60 third-year dental students. For educational purposes, a total of 61 anatomical regions and landmarks in both jaws, 20 intraosseous pathologies, and 26 dental materials with distinct radiographic appearances were identified. The educational intervention consisted of a self-paced, two consecutive 30-min interactive session in which students accessed the application via their mobile devices. During this session, participants navigated high-resolution radiographic images featuring interactive labels and descriptive annotations. Two 15-question surveys were developed in accordance with the learning content—one administered before and the other after the intervention. These surveys were delivered through the PanYoda platform. The normality of paired differences was assessed using the Shapiro–Wilk test. As normality assumptions were not met, pre- and post-training scores were compared using the Wilcoxon signed-rank test. Learning gain, normalized learning gain (Hake g) was calculated to quantify the magnitude of educational improvement. A *p*-value of < 0.05 was considered statistically significant.

**Results:**

The mean pre-training score increased from 65.92 ± 2.13 and 80.33 ± 2.21 after the educational intervention, yielding an average learning gain of 14.01 percentage points. The normalized learning gain indicated moderate improvement (g = 0.41). The Wilcoxon effect size was large (*r* = 0.87), suggesting a substantial difference between pre- and post-training scores. The improvement was statistically significant (*p* < 0.01).

**Conclusions:**

The findings suggest that the mobile-based educational application was associated with improved short-term radiographic interpretation performance. By providing interactive and immediate access to reference data at the point of care, the platform addresses some limitations of conventional analog and desktop-based resources and may serve as an effective and accessible supplement to contemporary dental education.

**Trial registration:**

Not applicable.

**Supplementary Information:**

The online version contains supplementary material available at https://doi.org/10.1186/s12909-026-09973-3.

## Background

Panoramic radiography is an imaging technique that visualizes the dental and maxillofacial regions of individuals in a single plane, providing additional morphological and pathological information not available in intraoral radiographs. It is considered an indispensable component of modern dentistry [1, 2]. Radiographic examinations are a vital part of diagnosis and treatment planning in daily clinical practice. Since the interpretation of radiographic images is typically performed by dentists themselves, the skills required for image reporting are taught during dental education, and panoramic radiographs (OPG) are the most frequently used imaging modality in clinical settings [3].

At the end of the clinical training in Oral and Maxillofacial Radiology at Erciyes University, third-year dental students are expected to acquire the following competencies: 1. Practical application of extraoral and intraoral examinations, 2. Practical application of routine dental and medical tests, 3. Interpreting OPGs, acquiring and interpreting intraoral radiographs, 4. Conducting clinical/radiographic examinations on patients [4]. As indicated in items 3 and 4, the ability to read and interpret radiographs is a mandatory skill for graduation. Moreover, the ability to read radiographs is essential throughout a dentist’s career to ensure accurate diagnoses and appropriate treatments.

However, several studies have shown that students often experience difficulties in accurately identifying anatomical landmarks and pathological findings on OPGs. Previous research has reported relatively low accuracy rates and high error rates in OPG interpretation among dental students [5–7]. These findings suggest that conventional educational approaches may not be sufficient to support the development of radiographic interpretation skills. With the advancement of digital technologies and artificial intelligence, access to information has become easier; however, verifying the reliability of accessed information remains essential [8]. One application developed to ensure access to accurate information is the web-based tool “PantoDict” [9]. However, most existing digital resources are limited by language barriers, outdated user interfaces, or a lack of comprehensive integration of localized clinical findings. To address these limitations, there is a need for a specialized, language-specific platform that aligns with the national dental curriculum and provides high-speed mobile accessibility.

Clinically, improving the ability to interpret OPGs may contribute to more accurate diagnosis and better treatment planning in daily dental practice. The null hypothesis of this study was that the mobile-based web application would not produce a significant difference in students’ OPG interpretation scores. The aim of this study is to evaluate the contribution of a newly developed, comprehensive Turkish-language web application designed to enhance the diagnostic accuracy and learning efficiency of third-year dental students.

## Methods

### Protocol and ethical principles

This study was designed as a single-group pre–post educational study and was approved by the Erciyes University Clinical Research Ethics Committee (2024/99) and conducted in accordance with the principles outlined in the Declaration of Helsinki. Furthermore, this research was conducted as an undergraduate student research project supported under the 2209-A Program by the Scientific and Technological Research Council of Turkey (TÜBİTAK) (Project No: 1919B012303369).

### Selection of the study group

Sixty third-year dental students (D3s) enrolled at the Faculty of Dentistry, Erciyes University, during the 2023–2024 academic year were included in the study. Inclusion criteria were enrollment as a third-year dental student, attendance in the relevant radiology coursework, and voluntary participation in the study. Students who did not complete both the pre- and post-training assessments were excluded from the analysis.

### Development of the web-based application

**Fig. 1:**
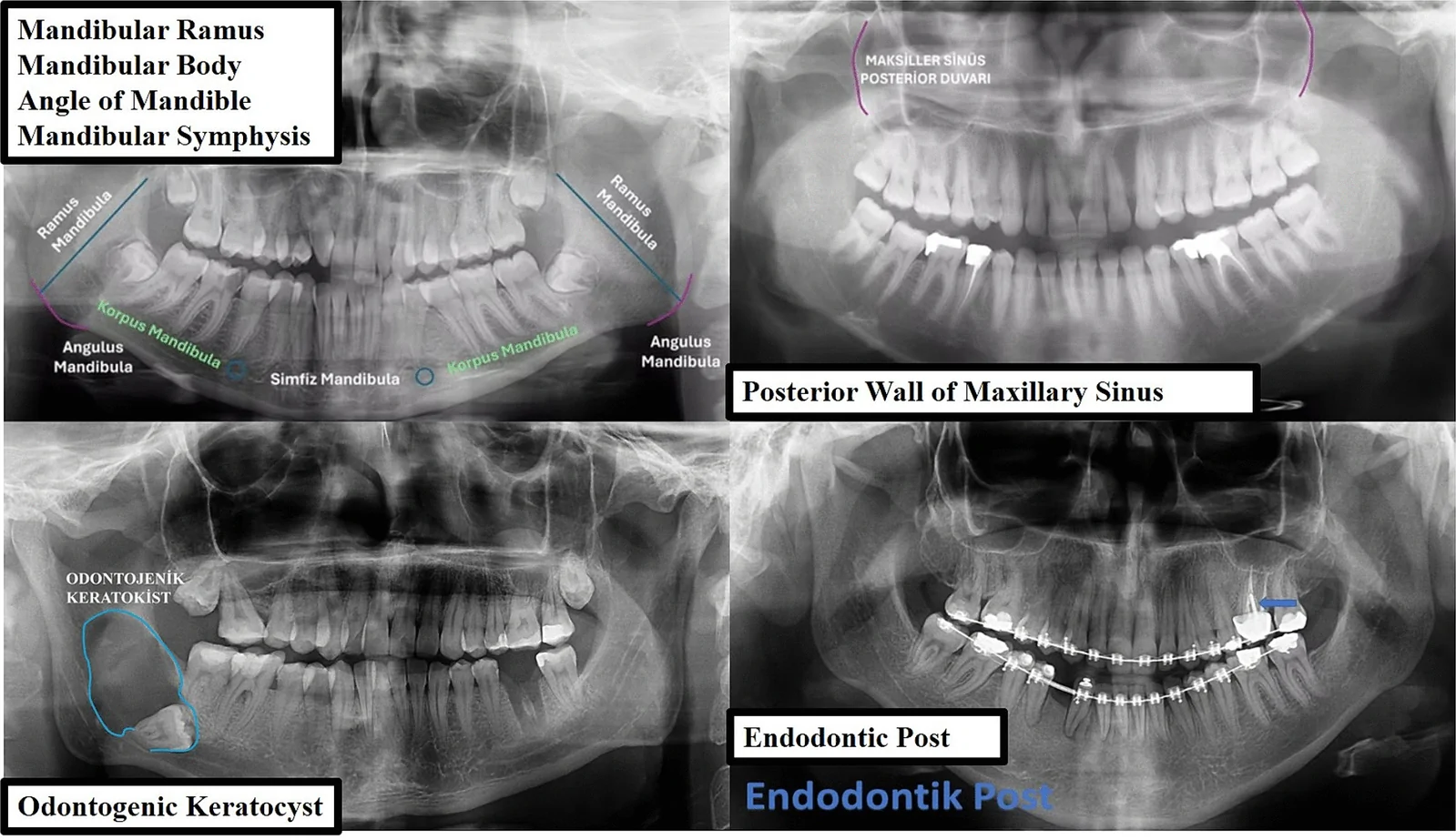
Display of the anatomical regions and landmarks, intraosseous pathologies, and dental materials selected for the training process

In the initial phase, a dataset of 219 high-quality OPGs was collected. From this dataset, 61 anatomical regions and landmarks, 20 intraosseous pathologies, and 26 dental materials were identified. These features were annotated on the OPGs by two researchers with clinical experience in oral and maxillofacial radiology to ensure visual clarity (Fig. 1). The selected images met predefined criteria, including high diagnostic quality and clear visibility of relevant structures. The annotated content was then integrated into a mobile-compatible, web-based application developed using the Wix platform [10, 11]. The final dataset was uploaded to the PanYoda system and made accessible to third-year dental students [10].

#### Educational intervention and survey preparation

**Fig. 2:**
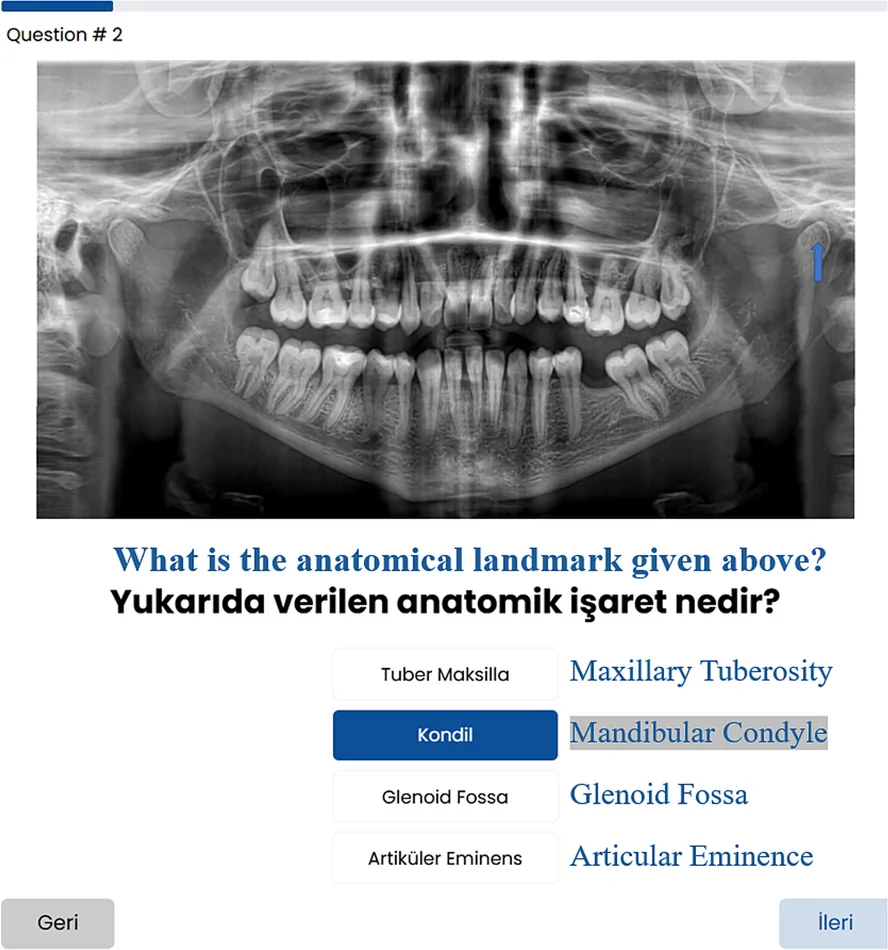
Screenshot of an example question from the survey applied through the PanYoda web-based application

A 15-question pre-training survey was developed based on the learning content (Fig. 2). After completing the pre-test, students participated in two consecutive 30-min interactive sessions. During these sessions, they used the mobile platform to review annotated OPG images. To assess knowledge gain, a post-training survey consisting of 15 different questions was administered. Both surveys covered the same content domains (anatomical landmarks, pathologies, and dental materials) with a balanced distribution of questions. The final version of the questionnaire was established through consensus between two experienced faculty members. Item difficulty and discrimination analyses were not performed and should be considered in future validation studies. Different radiographic cases and questions were used in the post-test to minimize recall bias. However, formal equivalence testing between assessments was not performed.

#### Preparation of the survey and data collection

The survey instrument was specifically developed for this study and implemented as an online quiz on the PanYoda platform (https://www.panyoda.com/quiz). The English version of the questionnaire is provided in Supplementary File 1. Each survey consisted of 15 multiple-choice questions, with four options and one correct answer. The surveys were integrated into the platform’s database, and scores were calculated automatically. Each correct answer was assigned 6.7 points, while incorrect or unanswered questions received 0 points, resulting in a total score range of 0–100.

#### Data validation

To ensure the validity of the assessment tool, the survey questions were reviewed and verified by two experienced faculty members in the Department of Oral and Maxillofacial Radiology. The digital records were then exported directly from the application's backend to a spreadsheet for statistical analysis, eliminating manual entry errors.

### Statistical analysis

Statistical analyses were performed using IBM SPSS Statistics version 22.0. Survey scores were treated as continuous variables, ranging from 0 to 100, based on the number of correct responses. The normality of paired differences was assessed using the Shapiro–Wilk test. As the assumption of normality was not met, pre- and post-training scores were compared using the Wilcoxon signed-rank test. Effect size was calculated as *r* = Z/√N.

**Fig. 3:**
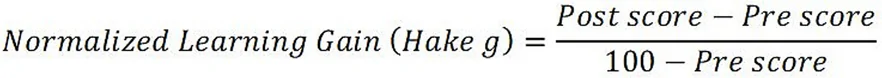
Normalized learning gain (Hake g) formula used to evaluate relative learning improvement following the educational intervention

To evaluate learning improvement, learning gain was calculated as the difference between post-training and pre-training mean scores. Normalized learning gain (Hake g) was also calculated to account for baseline performance (Fig. 3).

Trial registration: Not applicable.

## Results

Of the 60 D3s included in this study, 25 were female and 35 were male. The mean age of the participants was 21.58 (Table 1).

**Table 1:** Demographic characteristics of the participants

Gender	n	Age (x̄ ± Sd)
Female	25	21,16 ± 0,47
Male	35	21,26 ± 0,56
Total	60	21,22 ± 0,52

*n* Sample size, *x̄* Arithmetic mean, *SD* Standard deviation

The mean pre-training score was 65.92 ± 2.13, whereas the mean post-training score was 80.33 ± 2.21. Post-training scores were significantly higher than pre-training scores (Wilcoxon signed-rank test, Z = −6.748, *p* < 0.001). The effect size was large (*r* = 0.87), indicating substantial short-term improvement following the intervention. The normalized learning gain (g) was calculated as 0.41, suggesting a moderate level of relative improvement.

The improvement in scores was statistically significant (*p* < 0.01), suggesting an association between the intervention and improved short-term radiographic interpretation performance (Table 2).

**Table 2:** Pre- and post-training survey scores of the participants

Gender	Pre-Training (x̄ ± Sd)	Post-Training (x̄ ± Sd)	*p*	Test Statistic	Effect Size	Hake g
Female (n:25)	67,9 ± 1,50	81,16 ± 2,29	**< 0,001**	**-**	**-**	**-**
Male (n: 35)	64,5 ± 1,1	79,74 ± 1,99	**< 0,001**	**-**	**-**	**-**
Total (n: 60)	65.92 ± 2.13	80,33 ± 2,21	**< 0,001**	**Z = −6.748**	***r* = 0.87**	**0.41**

*x̄* Arithmetic mean, *Sd* Standard deviation, *p* Significance level, *Z* Wilcoxon signed-rank test statistic, *r* effect size calculated as Z/√N., *g* Normalized learning gain

Bold values indicate statistically significant results (*p* < 0,001)

## Discussion

The ability to read and interpret OPGs is a core competency required of all dentists. White and Pharoah proposed a systematic approach for radiographic evaluation, emphasizing the sequential assessment of features such as location, size, shape, borders, internal structure, and effects on surrounding structures [12]. In contemporary dentistry, the focus has shifted from comparing conventional and digital imaging systems to optimizing the educational and clinical use of digital technologies. Although earlier studies highlighted the advantages of digital systems in terms of workflow and image quality [13, 14], current evidence suggests that their primary value lies in facilitating interactive learning and rapid access to information. Furthermore, the effectiveness of radiographic interpretation is influenced not only by image quality but also by the observer’s experience and the capabilities of the application used [6, 15, 16, 17].

Previous studies have consistently demonstrated that students face challenges in interpreting OPGs. Razmus et al. reported low accuracy rates for anatomical landmarks and linked performance to clinical exposure [6]. Similarly, Maeda et al. found an overall accuracy rate of 53% and highlighted specific anatomical structures that are frequently misinterpreted [5]. Other studies have reported moderate success rates and substantial error rates among dental students when evaluating radiographic findings [7, 18]. These findings emphasize the need for more effective and accessible educational tools in radiology training.

Digital learning tools have been proposed as a solution to these challenges. For example, Bock et al. demonstrated that a web-based learning platform (PantoDict) improved students’ performance in OPG interpretation [9]. The platform evaluated in the present study differs from previous approaches by offering a mobile-first, point-of-care design that enhances accessibility and usability. By enabling students to access annotated radiographic images and reference data in real time, the application may support more efficient and flexible learning. This feature is particularly relevant in clinical settings, where immediate access to reliable information is essential [19].

The findings of the present study indicate a clear improvement in students’ performance following the intervention. The observed learning gain and moderate normalized gain suggest that the application was associated with improved short-term radiographic interpretation performance on the post-training assessment. In addition, the large effect size indicates a substantial difference between pre- and post-training scores. However, this effect size should be interpreted with caution, as it may be influenced by low variability in the data and the use of a single-group pre–post design. Therefore, while the results suggest a positive educational impact, they should be considered within the limitations of the study design.

Other digital approaches, such as tablet-based learning and virtual reality (VR), have also shown potential in radiology education. For instance, Tadinada et al. reported no significant difference between tablet and monitor-based evaluations, supporting the use of mobile devices in clinical practice [20]. Similarly, VR-based learning has been associated with improved anatomical understanding compared to traditional methods [21]. However, many of these approaches focus on procedural or immersive learning rather than the systematic interpretation of static radiographic images [22]. In this context, the present application provides a targeted approach that supports structured analysis of anatomical landmarks and pathologies.

Despite these promising findings, several limitations should be acknowledged. The use of a single-group pre–post design limits the ability to attribute the observed improvement solely to the intervention. Additionally, participants used personal mobile devices, which may have introduced variability in viewing conditions and potentially influenced radiographic interpretation due to differences in screen size, resolution, and brightness. The assessment instrument, although reviewed by experts, did not undergo formal internal consistency analysis (e.g., Cronbach’s alpha) or pilot testing, which should be considered a limitation. Furthermore, although the pre- and post-tests covered the same content domains, complete equivalence in difficulty between the two assessments cannot be guaranteed. Additionally, no formal assessment of inter-rater reliability between the experts was performed, which should be considered a limitation. Finally, the assessment focused on short-term outcomes, and long-term knowledge retention was not evaluated. Future studies should include controlled study designs, standardized display conditions, and longitudinal follow-up assessments to better evaluate the educational impact of such applications.

## Conclusions

In conclusion, this study demonstrates that the mobile-compatible educational application is associated with improved short-term radiographic interpretation performance. Its primary contribution lies in bridging the gap between theoretical knowledge and clinical practice through a mobile-optimized interface that enables immediate access to high-quality reference data at the point of care. By providing a more interactive and accessible alternative to conventional desktop-based tools and traditional analog resources, the platform represents an evidence-based adjunct to contemporary dental education and may serve as a useful educational supplement in dental education.

## Supplementary Information

Supplementary Material 1.

## Data Availability

The datasets generated and/or analyzed during the current study are available from the corresponding author on reasonable request.

## Abbreviations

OPG: Panoramic radiographs
D3s: Third-year dentistry students
g: Gain
UI: User interface
VR: Virtual reality
