# Supplementary material for: Does a mobile interactive web application improve dental students’ panoramic radiograph interpretation skills? A pre–post educational study
Source: BMC Med Educ. 2026 Jul 25;26:1693. doi: 10.1186/s12909-026-09973-3 (PMC13688731; doi:10.1186/s12909-026-09973-3)
Supplement: Supplementary file 1 [file 12909_2026_9973_MOESM1_ESM.docx]

**Supplementary File 1 – Survey Questionnaire Used in the Study**

**Title of Study:**

Impact of a Mobile Interactive Web Application on Dental Students’ Panoramic Radiograph Interpretation Skills: A Pre–Post Educational Study

**Description:**

This supplementary file presents the questionnaire used in the study. The survey instrument was specifically developed for this research and implemented as an online quiz on the PanYoda web platform. The survey consisted of 15 multiple-choice questions designed to evaluate students’ knowledge of anatomical landmarks, intraosseous pathologies, and dental materials on orthopantomographic images (OPGs).

Each question included four answer options with a single correct answer.

**Question 1**

What is the anatomical landmark shown above?

A) Maxillary Tuberosity

B) Mandibular Condyle

C) Glenoid Fossa

D) Articular Eminence

**Correct Answer: B**

**Question 2**

What is the anatomical landmark shown above?

A) Zygomatic Arch

B) Sigmoid Notch

C) Mandibular Coronoid Process

D) Pterygomaxillary Fissure

**Correct Answer: C**

**Question 3**

What is the anatomical landmark shown above?

A) Mental Foramen

B) Periapical Lesion

C) Mandibular Canal

D) Epiglottis

**Correct Answer: A**

**Question 4**

What is the anatomical landmark shown above?

A) Crown Fracture

B) Dental Filling Restoration

C) Dental Caries

D) Impacted Third Molars

**Correct Answer: D**

**Question 5**

What is the anatomical landmark shown above?

A) Styloid Process

B) Hamular Notch

C) Hyoid Bone

D) Pterygomaxillary Fissure

**Correct Answer: D**

**Question 6**

What are the anatomical landmarks shown above?

A) Mandibular Canal-Hyoid Bone

B) Mandibular Canal-Vertebrae

C) Mandibular Angulus-Epiglottis

D) Mental Foramen-Epiglottis

**Correct Answer: A**

**Question 7**

What is the anatomical landmark shown above?

A) Carotid Artery

B) Sigmoid Notch

C) Cervical Vertebrae

D) Thyroid Cartilage

**Correct Answer: C**

**Question 8**

What is the anatomical landmark shown above?

A) Ear Lobe

B) Sigmoid Notch

C) Zygomaticotemporal Suture

D) Zygomatic Arch

**Correct Answer: D**

**Question 9**

What are the anatomical landmarks shown above?

A) External Auditory Meatus -Periapical Lesion-Endodontic Post

B) Basion-Periapical Lesion-Dental Caries

C) External Auditory Meatus -Periapical Lesion- Laminate Veneer

D) External Auditory Meatus-Remaining Root-Dental Caries

**Correct Answer: D**

**Question 10**

What is the anatomical landmark shown above?

A) Endodontic Post

B) Implant Treatment

C) Dental Restoration

D) Mini Implant (TAD)

**Correct Answer: A**

**Question 11**

What is the anatomical landmark shown above?

A) Enamel Caries

B) Secondary Caries

C) Dental Restoration

D) Overfilled Restoration

**Correct Answer: B**

**Question 12**

What is the anatomical landmark shown above?

A) Cortical Bone

B) Soft Tissue Profile

C) Intraoral Structures

D) Horizontal Bone Loss

**Correct Answer: D**

**Question 13**

What is the anatomical landmark shown above?

A) Endodontic Post

B) Peri-implantitis

C) Mini Implant (TAD)

D) Periodontitis

**Correct Answer: B**

**Question 14**

What is the anatomical landmark shown above?

A) Mini-plate

B) Orthodontic Brackets

C) Lingual Retainer

D) Elastic Ligature

**Correct Answer: C**

**Question 15**

What is the anatomical landmark shown above?

A) Chronic Apical Periodontitis

B) Dentigerous Cyst

C) Lateral Periodontal Cyst

D) Endodontic File

**Correct Answer: A**
